# Organ Segmentation with Machine Learning Models

**DOI:** 10.3390/jimaging12080335

**Published:** 2026-07-24

**Authors:** Alexandros Barmperis, Olga Menegaki, Anna Panagiotakopoulou, Andreas Vezakis, Ioannis Vezakis, Ioannis Kakkos, George K. Matsopoulos

**Affiliations:** 1 Biomedical Engineering Laboratory, School of Electrical & Computer Engineering, National Technical University of Athens, 15773 Athens, Greece; 2 Archimedes Research Unit, Athena Research Center, 15125 Athens, Greece; 3 Department of Biomedical Engineering, University of West Attica, 12243 Athens, Greece

**Keywords:** abdominal CT segmentation, AMOS dataset, per-organ evaluation, nnU-Net, U-Mamba, Vision Transformer, state–space models, Hausdorff Distance, Normalised Surface Dice, deep learning benchmark

## Abstract

Accurate segmentation of abdominal organs in Computed Tomography (CT) underpins radiotherapy planning, surgical planning, and disease monitoring. Existing benchmarks rank architectures by a single aggregate Dice score, without per-organ statistical testing or boundary-sensitive metrics, even though models are chosen organ by organ for clinical use. We benchmark ten architectures spanning convolutional, attention-based, transformer, and state–space (Mamba) families on the AMOS CT dataset under one identical nnU-Net-style pipeline; we report per-organ Dice, 95-percentile Hausdorff Distance (HD95), and Normalised Surface Dice, with pairwise significance tested on an independent external dataset (TotalSegmentator). A competitive cluster of convolutional and Mamba models leads; rankings are stable on large organs but reshuffle by 10–13% on the small, geometrically complex ones, and boundary fidelity separates the models into tiers that the Dice ranking hides. This ordering largely holds on the external set (Spearman ρ=0.84). Selecting a model on aggregate Dice alone is therefore unsafe for organ-specific clinical tasks: per-organ overlap and boundary metrics should be the primary acceptance criteria for selecting a model before clinical deployment.

## 1. Introduction

Accurate segmentation of abdominal organs in Computed Tomography (CT) is a routine component of modern clinical workflows. In oncology, accurate delineation of organs-at-risk is the first step of radiotherapy planning: target volumes have to be defined precisely, and healthy organs have to be protected from excessive dose [1]. In surgery, three-dimensional organ maps help clinicians plan procedures, anticipate intra-operative challenges, and reduce operative risk [2]. For long-term disease monitoring, volumetric measurements of the liver and the spleen track conditions such as cirrhosis, portal hypertension, and abdominal malignancies [3]. In all of these settings, the accuracy of the downstream task is bounded by the accuracy of the underlying segmentation, which makes the choice of segmentation model a clinical decision rather than a purely methodological one.

Deep learning is now the standard tool for this task [4]. Convolutional Neural Networks [5,6,7,8], and in particular the self-configuring nnU-Net pipeline [9], remain a strong baseline. Vision Transformers [10] have since been adapted to volumetric medical data through models such as UNETR [11], Swin UNETR [12], and nnFormer [13], offering global context modelling at the cost of quadratic compute. State–space models such as U-Mamba [14] and SegMamba [15] have more recently proposed a linear time alternative. High-coverage pipelines such as TotalSegmentator [16] now annotate more than a hundred anatomical structures in CT.

The dominant practice is to report a single aggregate score, most often the mean Dice Similarity Coefficient (DSC) averaged across all organs [17,18]. The DSC is volume-weighted: it is dominated by large, well-defined structures and is largely insensitive to errors on small organs, where a handful of misclassified voxels can move the per-organ score by tens of percentage points [19]. In our study, we observe this asymmetry directly. The per-organ Dice on the liver and on the kidneys differs by less than 3%; on the gallbladder, the oesophagus, and the duodenum, it differs by more than twenty. A single mean Dice flattens that gap into a few decimal places and can rank a model that fails on the adrenal glands above a model that does not. The structures it under-weights are the small, geometrically complex, and pathologically variable ones where segmentation errors actually carry clinical consequences. Moreover, the Dice coefficient measures volumetric overlap, even a per-organ score can remain high on a large organ whose predicted boundary is clinically misplaced, since a thin surface error barely changes the overlapped volume. Overlap-based metrics, whether aggregated or per organ, are therefore incomplete on their own.

The Multi-Modality Abdominal Multi-Organ Segmentation (AMOS) dataset [20] is well suited to closing this gap. It provides expert annotations across fifteen abdominal organs that span roughly two orders of magnitude in volume, acquired across multiple scanners and centres. The recent comparison of state-of-the-art architectures on AMOS by Isensee et al. [18] reports only the mean Dice across the fifteen classes, leaving the per-organ behaviour of each model undocumented. Concretely, the original AMOS benchmark [20] and nnU-Net Revisited [18] approaches each report a single aggregate Dice value per model, averaged across all fifteen organs; neither reports organ-level Dice, HD95, or NSD individually, nor do they test whether the differences between architectures at the organ level are statistically significant.

This work is a comparative benchmark, not a new architecture. Our aim is to establish whether the choice of segmentation model can be made reliably from the evidence current practice reports. We make four contributions. First, we benchmark ten representative 3D architectures (convolutional and residual CNNs, attention-augmented CNNs, transformer encoders, and state–space (Mamba) hybrids) on AMOS CT under one identical nnU-Net-style pipeline, so that measured differences reflect the architecture rather than the training recipe. Second, we report every one of the fifteen organs individually under an overlap metric (Dice), two boundary metrics (95-percentile Hausdorff Distance and Normalised Surface Dice), and precision and recall, and show that the per-organ and boundary rankings depart from the aggregate Dice ordering. Third, we validate the ranking zero-shot on the independent TotalSegmentator dataset [16], determining whether the AMOS conclusions transfer off-distribution. Fourth, we assess pairwise significance with a valid per-case paired test on that external set. Unlike cross-validated predictions, every model is a fixed ensemble evaluated on identical cases. Together these establish which architectures are genuinely competitive, and on which organs and metrics that verdict changes.

## 2. Related Works

The Multi-Modality Abdominal Multi-Organ Segmentation (AMOS) benchmark [20] consolidates 500 CT and 100 MRI volumes across 15 abdominal organs, acquired from eight scanners across two clinical centres. It follows the line of multi-task public segmentation benchmarks established by the Medical Segmentation Decathlon [21], but concentrates a wide range of organ sizes into a single dataset. AMOS therefore exposes each architecture to the full spectrum of class-imbalance levels that abdominal CT presents. The original AMOS report benchmarks six baseline architectures and presents results as the mean Dice score averaged across the fifteen organ classes.

The most recent benchmarking on AMOS is the nnU-Net Revisited study of Isensee et al. [18]. Under a unified validation protocol, the authors compare CNN-, Transformer-, and Mamba-based segmentation networks across six datasets. They identify AMOS as one of the three most informative datasets in terms of inter- versus intra-method signal-to-noise, and report that a residual encoder nnU-Net achieves the best overall performance. The reported tables, however, again aggregate over the fifteen organ classes. Per-organ stratification on AMOS remains an open question across the benchmarks that have established it as a reference dataset.

### 2.1. Architectures

The U-Net family has dominated medical image segmentation since the introduction of the original 2D variant [6] and its V-Net [7] and 3D U-Net [8] volumetric counterparts, all of which build on the fully-convolutional segmentation methodology of Long et al. [5]. The self-configuring nnU-Net pipeline [9] makes these networks robust across datasets without manual tuning and remains a strong baseline. Its residual encoder variant [18] deepens the encoder with residual blocks. Both versions are evaluated in this work.

SegResNet [22] pairs an asymmetric encoder–decoder of residual blocks with a variational autoencoder branch [23] that reconstructs the input volume. The reconstruction loss regularises the shared encoder during training. The architecture won the BraTS 2018 challenge [24] and is widely used as a CNN baseline. Attention U-Net [25,26] is a closely related CNN variant. Originally proposed for pancreas segmentation, it inserts gated attention modules in the decoder that suppress irrelevant background activations.

MedNeXt [27] transfers ConvNeXt-style design back into a fully convolutional 3D encoder–decoder. It combines large depthwise kernels, inverted bottlenecks, and unified residual blocks for upsampling and downsampling. An iterative kernel upsizing scheme initialises a large-kernel model from a small-kernel one trained to convergence. The result is a pure convolutional network that reaches transformer-level performance on multiple medical benchmarks.

Vision Transformer [10] adaptations to volumetric data take three shapes. UNETR [11] pairs a pure ViT encoder with a CNN decoder. Swin UNETR [12] replaces global self-attention with hierarchical window-based attention to recover locality. nnFormer [13] interleaves local and global self-attention blocks with convolutional projections in a U-shaped network. MISSFormer [28] is a representative example of the broader convolution–transformer hybrid family. All four pay the quadratic cost of self-attention in sequence length, which is particularly heavy for 3D medical volumes.

State–space models provide a linear time alternative. Mamba [29] introduces a selective scan over the sequence dimension. U-Mamba [14] inserts a Mamba block either at the bottleneck (the Bottleneck variant) or after every encoder stage (the Encoder variant) of a residual U-Net, and both variants are evaluated here. SegMamba [15] extends the design with a tri-orientated scan over volumetric data. These models match transformer-style global context with a computational footprint closer to that of CNNs.

### 2.2. Per-Organ Evaluation Gap

Volumetric overlap, measured most often by the Dice Similarity Coefficient, has been the default segmentation metric for over a decade. Its main weakness is its volume weighting: a handful of misclassified voxels on a small organ produces a large drop in the per-organ score, while the same error on a large organ is invisible [19]. The Metrics Reloaded framework [17] argues that aggregate scores should be replaced by domain-aware metric selection driven by the structure of the task. The Normalised Surface Dice [30] complements volumetric Dice by measuring how often the predicted boundary falls within a clinically tolerable distance, and the 95-percentile Hausdorff Distance bounds the worst surface deviation in a robust way.

Despite this awareness, benchmark practice has not followed. TotalSegmentator [16], which labels 104 anatomical structures in CT, and the more recent AbdomenAtlas [31], an 8 500-volume abdominal CT dataset, both headline a single aggregate Dice. Per-organ tables, when available, are placed in the Supplementary Materials and rarely come with a significance test. A model that fails on the duodenum but excels on the liver can therefore occupy the same row of a leaderboard as a model with the opposite profile.

Table 1 places this study against the most directly comparable prior AMOS benchmarks. The pattern is consistent: earlier studies rank architectures by a single aggregate Dice, without per-organ statistical separation, boundary-tier (HD95) analysis, or external validation.

## 3. Materials and Methods

### 3.1. Dataset

We use the CT split of the Multi-Modality Abdominal Multi-Organ Segmentation (AMOS) dataset [20], which we obtained in its labelled training release of 300 CT volumes. The AMOS MRI subset is not used: restricting the benchmark to a single modality isolates architectural differences from the confounding effect of CT-versus-MRI intensity and preprocessing, and matches the CT modality of the external TotalSegmentator validation set. Each volume is annotated voxel-wise for fifteen abdominal organs: spleen, right kidney, left kidney, gallbladder, oesophagus, liver, stomach, aorta, inferior vena cava, pancreas, right adrenal gland, left adrenal gland, duodenum, bladder, and prostate/uterus. The volumes were acquired across two clinical centres and eight scanners, and span multiple contrast phases and disease states [20]. The unlabelled 200-volume test partition of AMOS is not used: we evaluate by internal 5-fold cross-validation rather than against the hidden challenge server, so that every model produces one prediction per volume under an identical evaluation protocol (Section 3.8).

The fifteen target organs span more than two orders of magnitude in volume, from the liver and the spleen at the upper end to the adrenal glands and the gallbladder at the lower end. This range is intentional: AMOS is one of the few public benchmarks that exposes a single architecture to the full spectrum of class-imbalance levels found in abdominal CT, which makes it appropriate for the per-organ analysis presented here.

**Figure 1:**
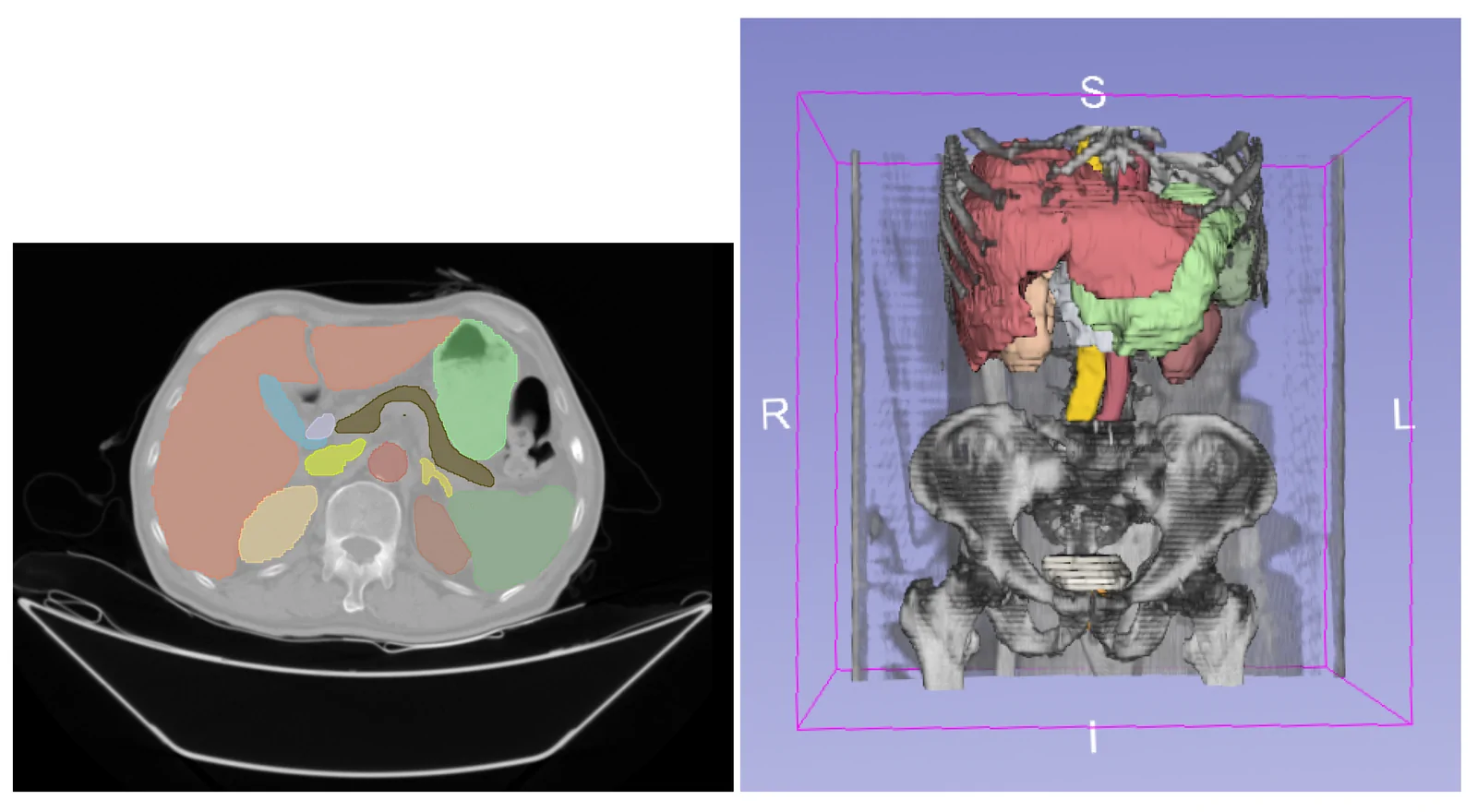
A single sample from the AMOS CT split. (**Left**): axial slice with per-organ overlay (colour-coded). (**Right**): 3D volume rendering with the segmentation visible through the body. Both panels were generated in 3D Slicer 5.10.0 [33].

**Figure 2:**
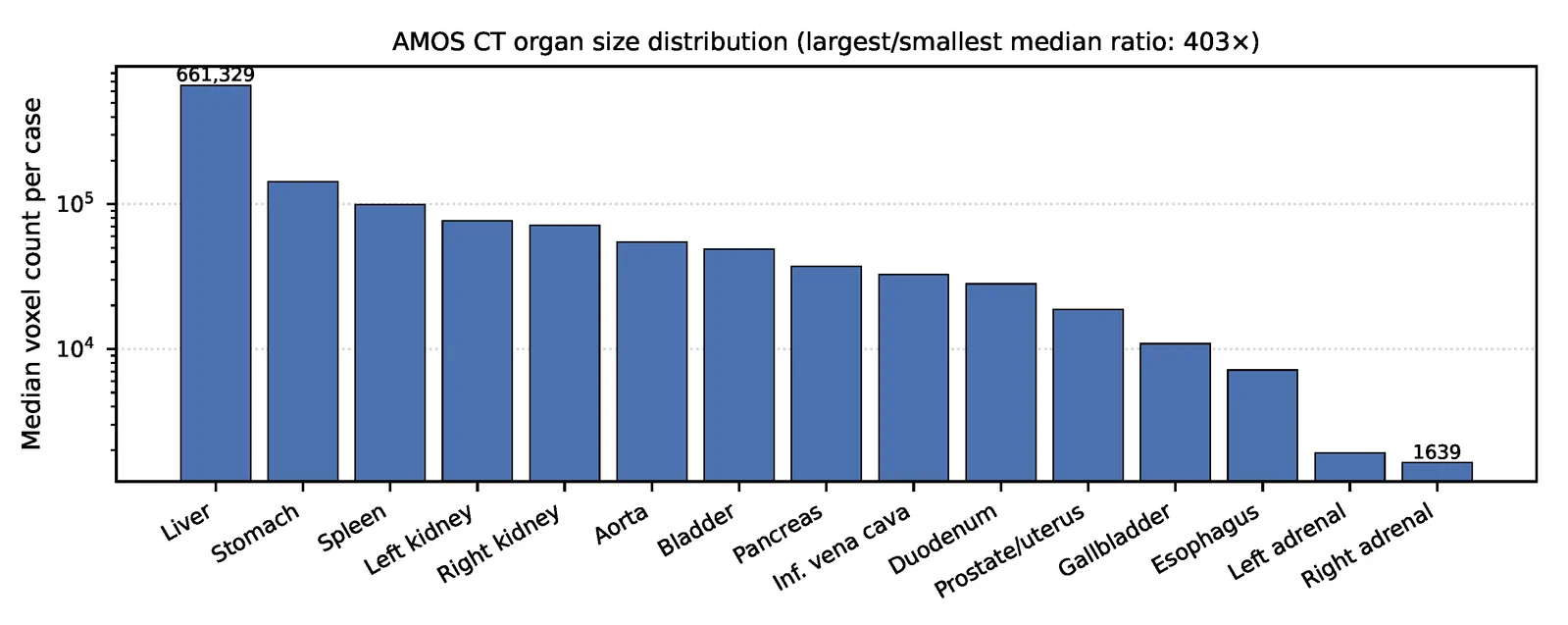
Per-organ size distribution on the AMOS CT split, illustrating the more than two orders of magnitude range that motivates the per-organ analysis.

The dataset was split into five folds with deterministic per-fold case lists, generated once and reused across every architecture (240 training and 60 validation cases per fold). Using the same splits across models ensures every model is evaluated on identical validation cases per fold, so the per-organ comparison rests on common data (Section 3.8). Figure 1 visualises one sample, and the relative organ sizes that drive the analysis are shown in Figure 2.

### 3.2. Preprocessing

Preprocessing follows the nnU-Net v2 self-configuring pipeline [9], which infers the target spacing, patch size, and intensity normalisation from a dataset fingerprint. This adaptive pipeline has become a de facto standard in 3D medical image segmentation, adopted or built upon well beyond nnU-Net itself, including by MedNeXt [27] and the U-Mamba variants [14] evaluated here. So applying it uniformly across all ten architectures reflects common practice rather than privileging any single model. Each volume is cropped to its nonzero foreground, intensity-normalised, and resampled to a target voxel spacing chosen by the planner. CT intensities are clipped to the foreground 0.5–99.5 percentile range estimated on the training set (approximately [−986,+379] HU) and then z-scored using the foreground mean and standard deviation. The same statistics are applied at the inference time.

Two preprocessing configurations are produced for AMOS:

- 3d_fullres (anisotropic). Target spacing 2.0×0.685×0.685 mm, patch size 64×192×192 voxels, median resampled volume 104×512×512 voxels.
- 3d_fullres_iso (isotropic). Target spacing 0.979×0.979×0.979 mm, patch size 128×128×128 voxels, median resampled volume 213×358×358 voxels.

The isotropic spacing is the geometric mean of the anisotropic target, so that the two configurations yield patches of comparable physical volume and voxel count; each network therefore processes approximately the same field of view, and the same amount of anatomical context, per patch, regardless of the underlying voxel grid. The dynamic-topology models are resolution-adaptive: the planner derives their patch size and pooling directly from the native voxel spacing. Forcing all ten models onto a single common grid would therefore pull these volume-adaptive architectures off the resolution they are designed for. This is equivalent to feeding anisotropic patches to models built for isotropic voxels, and would inject a new bias rather than remove the confound. Therefore, model is run under the configuration that matches its topology mode. Architectures with a fixed pooling layout (SegResNet, Swin UNETR, UNETR, nnFormer, MedNeXt, and the Attention U-Net configuration evaluated here) require isotropic voxels and therefore use 3d_fullres_iso. Architectures with a planner-derived dynamic topology (nnU-Net plain, nnU-Net ResEnc M, U-Mamba Bottleneck, U-Mamba Encoder) use 3d_fullres, matching the configuration used by their authors on AMOS. The configuration is the only experimental setting that varies between architectures; all other training and inference details are identical.

Because the fixed-topology models run on isotropic voxels and the dynamic-topology models on anisotropic voxels, a residual confound remains: any gap between the two groups reflects each architecture together with its compatible input resolution, not the architecture in isolation. We mitigate this by matching physical patch volume rather than the raw voxel grid, so that both groups see approximately the same anatomical field of view per patch; the confound is therefore bounded, not introduced by an arbitrary choice of resolution.

### 3.3. Models

The ten architectures sample the principal families of 3D medical image segmentation rather than enumerate every published network. Four criteria guided the selection: applicability to volumetric CT, a public and reproducible implementation, a documented record as a strong baseline or recent state-of-the-art model, and coverage of a distinct methodological family. Where a family is strongly represented in the literature, we include more than one member, so that a family-level trend is not read off a single implementation. Accordingly, nnU-Net in its plain and residual encoder [18] forms provides the self-configuring convolutional baseline [9]; SegResNet [22], Attention U-Net [25], and MedNeXt-B [27] add autoencoder-regularised residual, attention-gated, and ConvNeXt-inspired convolutional designs; UNETR [11], Swin UNETR [12], and nnFormer [13] represent transformer encoders spanning global, windowed, and interleaved attention; and the U-Mamba Bottleneck and Encoder variants [14] represent recent state–space hybrids. The aim is not to identify the single best model within any category, but to compare strong, reproducible representatives of each family under one identical protocol. Every model is instantiated from its official implementation with the architectural defaults of its source paper or codebase, unmodified. Table 2 summarises the ten models with their methodological family, topology mode, preprocessing configuration, and trainable parameter count, measured from the fold-0 checkpoint. Architectural descriptions for each family are given in the Section 2.

The nnU-Net ResEnc M variant is the “M” (medium-VRAM) preset introduced in nnU-Net Revisited [18], which deepens the encoder with residual blocks. SegResNet places its variational autoencoder branch on the encoder side, where the reconstruction loss regularises the shared encoder during training; the branch is dropped at inference. MedNeXt is evaluated at its B (base) size preset with kernel size three and the kernel-upsampling refinement disabled, for consistency with the other models. The two U-Mamba variants differ only in where the Mamba selective-scan block is inserted: between the encoder and the decoder (Bottleneck) or after every encoder stage (Encoder). Because the Mamba CUDA kernels are fp32-only, mixed precision is disabled for both U-Mamba variants; all other models run under the same default training precision.

### 3.4. Training

All ten models share a single training recipe modelled after the nnU-Net v2 trainer [9]. We use stochastic gradient descent with Nesterov momentum 0.99, weight decay $3\times 10^{-5}$, initial learning rate 0.01, and a polynomial decay schedule $\eta_{e}=\eta_{0}\;{\left(1-e/E\right)}^{0.9}$. The loss is the unweighted sum of a soft Dice loss (with background excluded and smoothing $10^{-5}$) and a cross-entropy loss. Deep supervision is used at all decoder stages that produce auxiliary outputs, with per-scale weights decaying as $1/2^{i}$ and renormalised to sum to one. Gradients are clipped at an $L_{2}$-norm of 12.

Each model is trained for E=1000 epochs of 250 training iterations and 50 validation iterations each, in the nnU-Net sense; an “epoch” is therefore a fixed iteration budget rather than a full pass over the dataset. Patches are sampled with foreground oversampling at 33% per batch. Augmentation follows the nnU-Net default suite: random rotations (probability 0.2), random scaling in [0.7,1.4] (probability 0.2), Gaussian noise (0.1), Gaussian blur (0.2), brightness scaling in [0.75,1.25] (0.15), contrast scaling in [0.75,1.25] (0.15), low-resolution simulation in [0.5,1.0] (0.25), gamma correction with normal and inverted variants (0.3 and 0.1, range [0.7,1.5]), and mirroring along every spatial axis. The same patch size and batch size are used for every fold of a given model, both taken from the planner’s choice for that model’s preprocessing configuration (a batch size of two in every case). Mixed precision is disabled in every run reported here to keep the loss landscape identical across architectures and to accommodate the fp32-only Mamba kernels.

The best checkpoint per fold is selected by the exponential moving average (smoothing factor α=0.9) of the foreground Dice on the validation iterations. Every run is executed on a single NVIDIA A100-SXM4-80GB GPU (NVIDIA Corporation, Santa Clara, CA, USA). All models are implemented in PyTorch (v2.11.0) and MONAI (v1.5.2), with experiment configuration managed by Hydra (v1.3.2). With ten architectures and five folds, the experimental matrix contains fifty training runs. The protocol above deliberately separates shared from model-specific settings. Every training-level hyperparameter (the optimizer and its momentum, the learning-rate schedule, the loss, the augmentation policy, and the deep-supervision weighting) is held identical across all ten architectures, so that the training recipe cannot itself confound the comparison. This is essential for fairness, because these choices affect accuracy independently of the network: letting one model use, say, a different optimizer, a longer schedule, or a tuned loss would advantage it for reasons unrelated to its architecture. What remains model-specific is limited to what is structural, since altering it would change the network itself. This includes MedNeXt-B’s kernel size and inverted bottleneck; the planner-derived patch size and topology of the four dynamic-topology models (nnU-Net plain, nnU-Net ResEnc M, and both U-Mamba variants); and the fixed pooling layout of the remaining six architectures. Each of these is kept at its published default.

### 3.5. Inference and Validation

Each of the fifty trained checkpoints is evaluated on the sixty held-out validation cases of its own fold. We do not produce a cross-fold ensemble. The per-fold validation Dice serves as the unit of comparison across architectures, matching the convention used in nnU-Net and its variants [9,18]. Because all ten models share the same fold definitions, the per-fold Dice statistics for any given organ describe the same underlying validation cases, and combining the five folds gives a per-organ summary over the full 300-case validation pool that underlies the descriptive analysis of Section 3.8.

Inference uses the nnU-Net v2 sliding-window predictor with 50% patch overlap and a Gaussian importance map at the patch borders. Patches are processed in batches of sixteen on the GPU. Test–time augmentation averages predictions over all subsets of spatial mirroring axes. Predictions are inverted through the preprocessing pipeline to return them to the original voxel grid before metric computation.

### 3.6. External and Cross-Dataset Validation

The AMOS ranking may not generalize beyond its own distribution. We test this directly by evaluating the trained models zero-shot on TotalSegmentator v2 [16], an independent, multi-institutional CT collection that annotates 104 anatomical structures over more than a thousand scans acquired on a broad range of scanners, reconstruction kernels, and contrast phases. It is assembled from different centres and protocols than AMOS, and its reference masks come from an automated (nnU-Net-derived) labelling pipeline rather than the AMOS expert protocol. It therefore presents two shifts at once, a covariate shift in image appearance and an annotation-convention shift. These are precisely the conditions under which a benchmark ranking is at risk of not transferring.

We draw 100 abdominal CT volumes from TotalSegmentator and align them to our setting without altering the models. The fifteen AMOS organs are recovered by remapping TotalSegmentator’s per-structure masks onto the AMOS fifteen-class label order (for example kidney_right→right kidney, adrenal_gland_left→left adrenal gland); structures absent from a case remain as the background, and are excluded from that case’s aggregation. Each volume is then preprocessed with the AMOS plans (the same foreground intensity clipping, normalisation statistics, and target spacing used in training) so that the external data reach each network exactly as its training distribution did, with no re-fingerprinting of the external set. Every model is run under the configuration it was trained with (isotropic or anisotropic), through the same frozen five-fold ensemble, sliding-window inference, and metric computation as on AMOS.

### 3.7. Metrics

Three primary metrics (overlap, boundary distance, and surface agreement) are computed per organ class on every validation volume, and three confusion-matrix metrics are reported alongside them.

The Dice Similarity Coefficient (DSC) measures the volumetric overlap between the predicted and the reference mask, expressed as DSC(P,G)=2|P∩G|/(|P|+|G|). We use the per-class formulation with the background excluded.

The 95-percentile Hausdorff Distance (HD95) measures the boundary distance between the predicted and the reference contours in millimetres, taking the 95-percentile of the two-sided point-to-surface distances. It is more robust to a small number of outlier voxels than the standard Hausdorff Distance and is commonly used for clinical boundary fidelity [17].

Normalised Surface Dice (NSD) measures the fraction of the predicted boundary that lies within a fixed tolerance of the reference boundary [30,34]. We use a uniform tolerance of 1.0 mm across all fifteen organs, in line with the default validation configuration of the nnU-Net evaluation script [9]. A uniform tolerance allows direct cross-organ comparison; an organ-specific tolerance derived from inter-rater variability would tighten the metric on small organs and is left as a limitation.

Precision, recall, and the Jaccard index decompose and complement the overlap score. Writing TP, FP, and FN for the true-positive, false-positive, and false-negative voxel counts of an organ, precision =TP/(TP+FP) and recall (sensitivity) =TP/(TP+FN) splits the overlap error into over- and under-segmentation, whereas the Jaccard index (intersection-over-union) =TP/(TP+FP+FN) is a stricter overlap measure monotonically related to Dice. These are reported as complementary evidence Dice, HD95, and NSD remain the primary comparison metrics.

All three primary metrics are aggregated in a fixed order: per (model, fold, volume, organ) values are averaged over the sixty cases within each fold to produce per-fold per-organ means, and the five per-fold means are then summarised as their mean and standard deviation. Every per-organ number reported in the Section 4 follows this aggregation, which is identical to the one used by the upstream nnU-Net validation script and to the cross-validation conventions in nnU-Net Revisited [18]. The precision, recall, and Jaccard index defined above follow the same per-organ aggregation.

### 3.8. Statistical Analysis

On AMOS we report results descriptively. For a given (model, organ), let $s_{f}$ denote the mean Dice over the sixty validation cases of fold *f*; we report the cross-fold mean and standard deviation

(1) $$\bar{s}=\frac{1}{5}\sum_{f=1}^{5}s_{f},\;\sigma =\sqrt{\frac{1}{5-1}\sum_{f=1}^{5}{\left(s_{f}-\bar{s}\right)}^{2}},$$

and rank and cluster models by these aggregates. We deliberately attach no pairwise significance test to the AMOS numbers. Under 5-fold cross-validation each of the 300 cases is segmented by the single fold whose validation split contains it, so a model’s prediction for a given case is produced by a different trained instance depending on the case. A paired test over the pooled 300 cases would therefore not compare two fixed models on a common sample; it would concatenate five distinct per-fold model comparisons and treat them as one. Testing per fold and then combining the five *p*-values only re-introduces a dependence problem, because any two folds’ models share three of their four training folds. There is no assumption-free paired test on cross-validated predictions.

We instead perform the paired significance test on the external TotalSegmentator set (Section 4.7). There, every model is a single fixed function (the frozen five-fold ensemble) evaluated on the identical n=100 cases, so for two models, *R* and *M*, the per-case Dice difference is as follows:

(2) $$d_{i}={\mathrm{DSC}}_{R}\left(x_{i}\right)-{\mathrm{DSC}}_{M}\left(x_{i}\right),\;i=1,\ldots ,n,$$

which is a genuine paired observation of a single comparison. Taking the top-ranked external model as the reference *R* and each other model as a challenger *M*, we test $H_{0}:\mathrm{median}\left(d_{i}\right)=0$ against the one-sided alternative R>M with the Wilcoxon signed-rank statistic:

(3) $$W^{+}=\sum_{i\;:\;d_{i}>0}\mathrm{rank}\;\left(|d_{i}|\right),$$

which is evaluated per organ on the cases in which that organ is present, and once on the per-case fifteen-organ mean Dice. Across the m=15 organs, the *p*-values are Holm–Bonferroni-corrected [35]: with ordered values $p_{\left(1\right)}\leq \ldots \leq p_{\left(m\right)}$, hypothesis $H_{\left(k\right)}$ is rejected while $p_{\left(k\right)}\leq \alpha /\left(m-k+1\right)$ at α=0.05, controlling the family-wise error rate. At n=100, even a negligible but consistent gap reaches significance; therefore, we report each comparison’s effect size (the median per-case difference $\tilde{d}={\mathrm{median}}_{i}d_{i}$ and the win rate $\omega =\frac{1}{n}\sum_{i}1\left[d_{i}>0\right]$) alongside the *p*-value. The Wilcoxon signed-rank test [36] is preferred over the paired *t*-test because per-case Dice is bounded and skewed, and is the standard choice for paired model comparison [37].

## 4. Results

Every model was trained from scratch on the AMOS CT split under the unified protocol of Section 3.3, and every per-organ number reported below follows the aggregation pipeline defined in Section 3.7: the per-fold means are calculated over the sixty validation cases, then the mean ± is the standard deviation of the five per-fold means. Tables and figures use the same model ordering throughout: descending overall Dice on AMOS.

### 4.1. Aggregate Performance

Table 3 reports the three primary metrics aggregated over the fifteen organs and the five validation folds. The residual encoder nnU-Net ranks first on Dice and on HD95, and leads nnU-Net plain by only 0.4 Dice points on overall Dice (0.893 vs. 0.889); the two are statistically inseparable on the external test set (Section 4.7). Attention U-Net follows at 0.885 Dice and is the top-ranked model on Normalised Surface Dice (0.826). The two U-Mamba variants share the next two positions at 0.879 Dice. Swin UNETR, MedNeXt-B, nnFormer, UNETR, and SegResNet trail the top cluster by 2 to 12%.

Three patterns follow: the top five rows on Dice are all CNN or CNN-attention hybrids, including the two transformer-only architectures (UNETR, nnFormer) and SegResNet trail. HD95 is not a rephrasing of Dice: UNETR and SegResNet have HD95 values three to four times worse than the leaders, while the Dice is only 10 to 12% behind. NSD picks a different top-ranked model than Dice (Attention U-Net edges the nnU-Net pair). These reshuffles foreshadow the per-organ behaviour examined next.

Beyond these three primary metrics, Table 4 reports precision, recall (sensitivity) and the Jaccard index (IoU), foreground-averaged and aggregated identically. Precision and recall decompose the overlap error that Dice summarises into false-positive (over-segmentation) and false-negative (under-segmentation) components. They are near-balanced for the convolutional and Mamba leaders, but diverge for SegResNet, whose recall (0.772) trails its precision (0.810), an under-segmentation case that the aggregate Dice hides.

### 4.2. Mean Dice Versus Model Size

**Figure 3:**
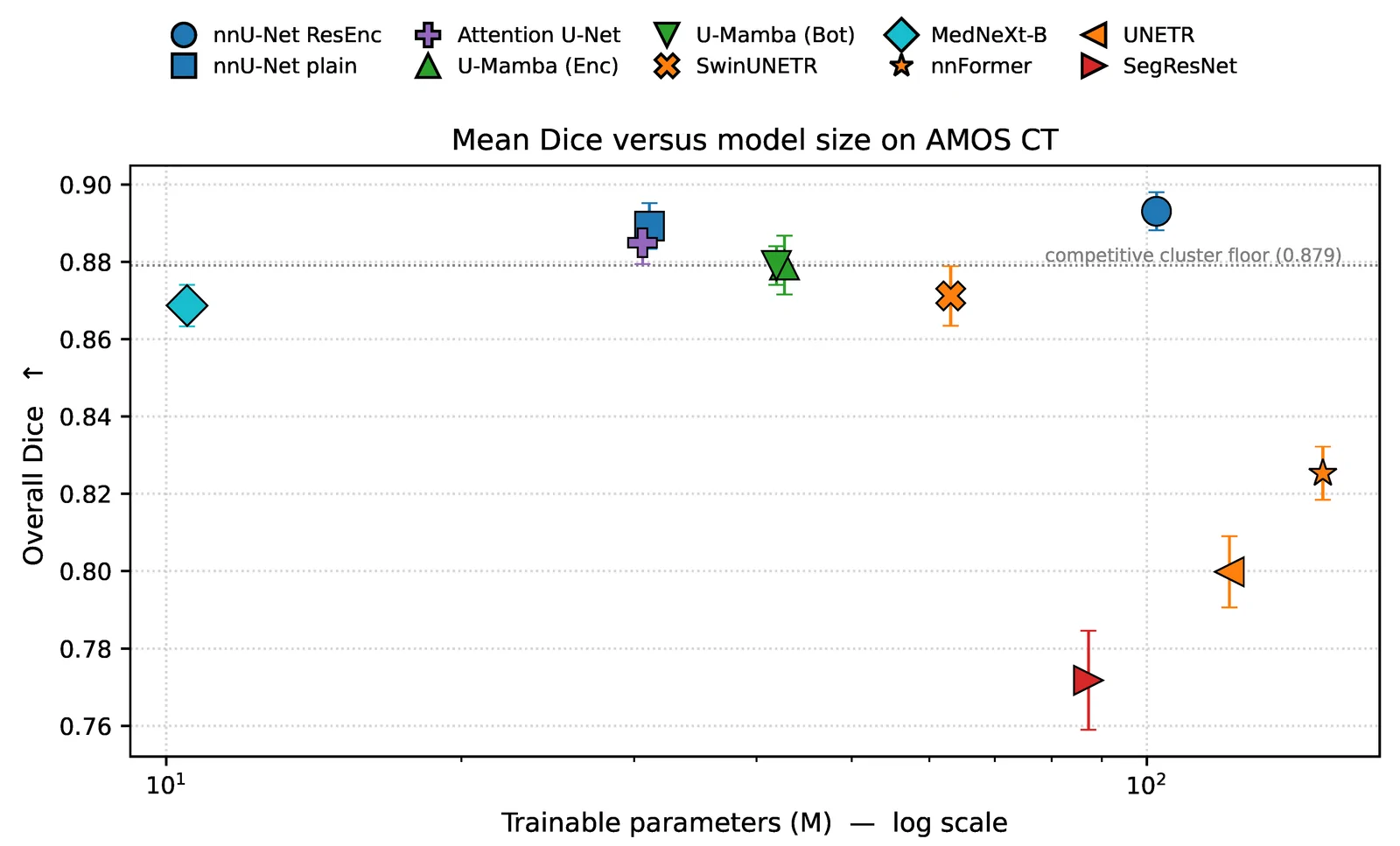
Overall Dice on AMOS CT versus trainable parameter count, one point per model. Error bars are the standard deviation across the five validation folds; the horizontal dotted line marks the lowest overall Dice within the top competitive cluster (0.879, the two U-Mamba variants). The relationship is non-monotonic and, in fact, weakly negative across the ten architectures (Pearson r=−0.52 on log-parameters; Spearman ρ=−0.39). MedNeXt-B (10.5 M, smallest) outperforms nnFormer (151 M, largest) on Dice by 4.4%.

Figure 3 plots overall Dice (with the cross-fold error bar of Table 3) against the trainable parameter count from Table 2. The relationship is not monotonic with size and, across the ten-model matrix, runs in the opposite direction from what scaling intuition would predict: the Pearson correlation between log-parameters and overall Dice is r=−0.52 (p=0.12, n=10), and Spearman ρ=−0.39. Three specific contrasts make the point. MedNeXt-B is the smallest model in the evaluation at 10.5 M parameters and yet beats every transformer-only architecture on overall Dice (0.869 vs. 0.825 nnFormer, 0.800 UNETR), each of which carries an order of magnitude more parameters. nnU-Net plain at 31 M matches nnU-Net ResEnc M at 102 M to within 0.4 Dice points on overall Dice—and is statistically inseparable from it on the external set (Section 4.7)—a 3.3-fold scaling that increases the Dice by 0.4%. nnFormer at 151 M, the largest model in the matrix, sits two Dice tiers below the leaders. At AMOS scale (300 labelled CT volumes), the dominant lever for overall accuracy is therefore architectural inductive bias, not parameter count [38,39]. The same conclusion echoes the per-organ analysis that follows, where the leaders consistently exploit a planner-derived convolutional topology rather than raw capacity. The compute and memory this capacity costs are quantified next (Section 4.3, Table 5).

### 4.3. Computational Efficiency

Parameter count alone does not determine deployment cost, which is set by arithmetic and memory. Table 5 adds three measured quantities to the parameter counts of Table 2: the floating-point operations of a single forward pass on one patch (GFLOPs), the wall-clock time of one full-volume sliding-window inference, and the peak GPU memory during that inference. All three are properties of the architecture at its AMOS input resolution and are independent of the trained weights; they were measured on a single NVIDIA A100-SXM4-80 GB.

Three patterns emerge. First, the computation is not predicted by size: UNETR holds the second-largest parameter count (121 M) but the second-lowest GFLOPs (353), because a plain ViT encoder spends its budget on wide token-projection matrices rather than spatial convolutions; in contrast, nnU-Net ResEnc reaches 2346 GFLOPs at a comparable parameter count and neither ordering matches the Dice ranking. Second, the accuracy–efficiency frontier belongs to the small convolutional models. nnU-Net plain equals the residual encoder leader on Dice (Table 3) at one-third the parameters, roughly half the GFLOPs, and 0.8 s less time per volume, so the residual encoder’s extra capacity buys no measurable accuracy at AMOS scale. MedNeXt-B, in turn, carries the fewest parameters and the fewest GFLOPs of any model, yet outscores both transformer-only architectures. Third, the state–space models are the most expensive at inference: U-Mamba (Enc), which inserts a selective-scan block after every encoder stage, needs 8.6 s and 12.4 GB per volume—this is nearly five times the time and over twice the memory of nnU-Net plain for the same overall Dice; in contrast, U-Mamba (Bot), with a single bottleneck scan, costs less than half as much. Wall-clock also decouples from FLOPs: MedNeXt-B has the lowest FLOP count but one of the higher inference times (5.0 s), reflecting the kernel-launch overhead of its many large-kernel depthwise blocks.

Because the two preprocessing configurations give each model approximately the same number of voxels per patch (Section 3.2), the inference time and peak memory columns are directly comparable deployment figures, while GFLOPs and parameters are the resolution-independent comparators. The training wall-clock is not tabulated: under the shared fixed-iteration schedule, relative training cost tracks the per-forward GFLOPs already reported, and the absolute wall-clock is dominated by shared data loading and the scheduling of the cluster used here.

### 4.4. Per-Organ Volumetric Performance

**Figure 4:**
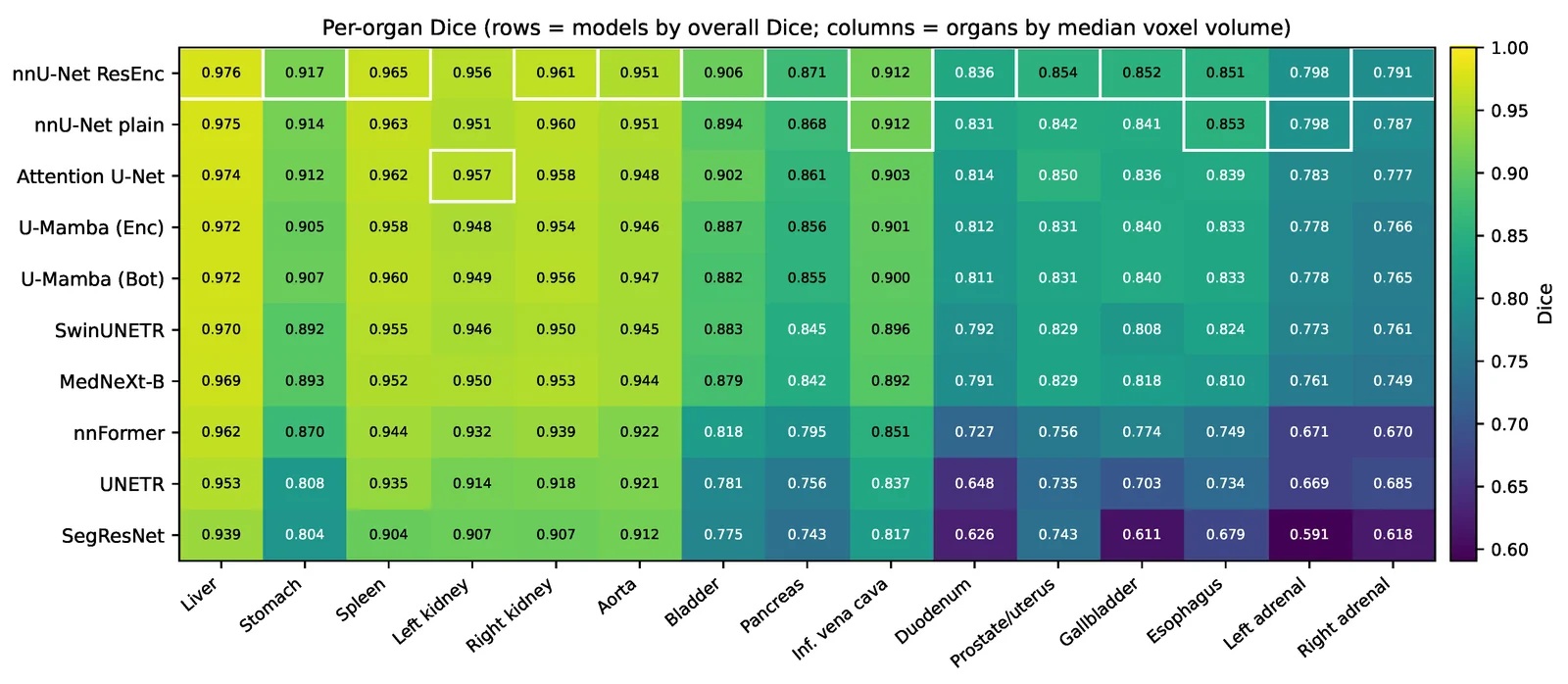
Per-organ Dice heatmap on AMOS. The within-column variation grows as organs shrink: the liver and the spleen are saturated across the competitive cluster, whereas the adrenal glands, duodenum, and oesophagus separate the architectures’ Dice 10–13% within the same competitive cluster (top eight models).

Table 6 reports the per-organ Dice for the ten models, and Figure 4 visualises the same data as a heatmap with organs ordered from largest to smallest. The single most informative pattern in the data is that the within-column variation grows monotonically as we move from the large, well-defined organs to the small, geometrically complex ones.

The large, high-contrast organs are saturated: across the top eight, the Dice spread is 1.4% on the liver, 2.1% on the spleen, and 2.2% on each kidney—below the resolution of the test. The spread grows with anatomical difficulty, reaching 10.3% on the oesophagus, 10.9% on the duodenum, and 12.2–12.7% on the adrenal glands. The smallest organs therefore separate the architectures by an order of magnitude more than the largest ones.

Per-organ leadership concentrates in the CNN family. nnU-Net ResEnc is top-1 on eleven of fifteen organs; nnU-Net plain is top-3 on every organ; Attention U-Net is top-3 on thirteen of fifteen. The Mamba and ConvNeXt-3D families are competitive but never lead an organ. The tail is consistent: SegResNet and UNETR occupy the bottom two rows on every organ, with single-organ failures below 0.65 Dice (SegResNet gallbladder 0.611, UNETR duodenum 0.648).

### 4.5. Boundary Fidelity

**Figure 5:**
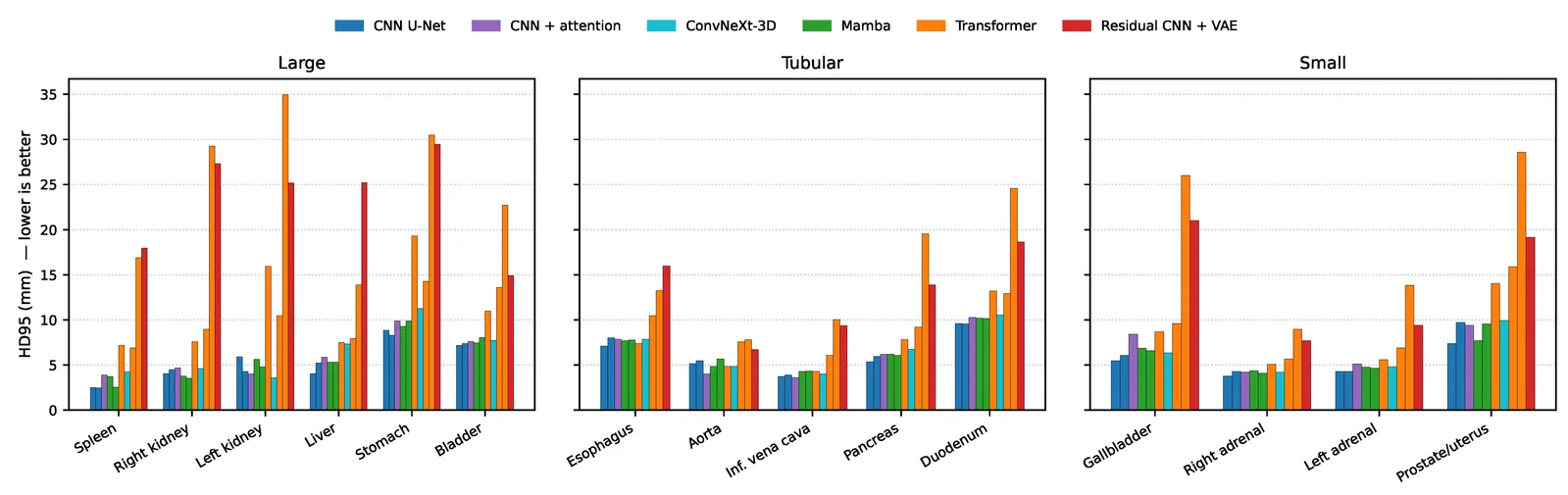
Per-organ HD95 grouped by organ size tier. SegResNet and UNETR fail catastrophically on most organs; the remaining six architectures cluster tightly inside the 5–10 mm band.

The HD95 table (Table 7) and the grouped bar chart in Figure 5 partition the ten models into three tiers with sharp gaps. The first tier sits at 5–7 mm overall HD95 and contains nnU-Net ResEnc, nnU-Net plain, U-Mamba Bot, U-Mamba Enc, Attention U-Net, and MedNeXt-B. The second tier at 9–10 mm contains Swin UNETR and nnFormer. The third tier, at 17–20 mm, contains UNETR and SegResNet. The inter-tier gaps are substantially larger than the corresponding gaps on Dice, which means HD95 separates the architectures more sharply than the volumetric metric.

Inside the top tier, the U-Mamba variants beat the CNN leaders on specific organs: U-Mamba (Bot) has the lowest HD95 of any model on the right kidney (3.5 mm vs. 4.0 mm) and is within 0.1 mm of the leader on the spleen. These boundary fidelity wins are invisible in the Dice ranking.

The transformer family struggles disproportionately on HD95. nnFormer’s HD95 is 1.5–3 times worse than the CNN tier on every organ despite being only one Dice tier behind, and UNETR has HD95 above 25 mm on five organs. This is the Dice-good/boundary-bad failure mode that Maier-Hein et al. [17] identify as the textbook reason why volumetric overlap is an insufficient evaluation.

### 4.6. Architectural Behaviour on Long-Tail Organs

**Figure 6:**
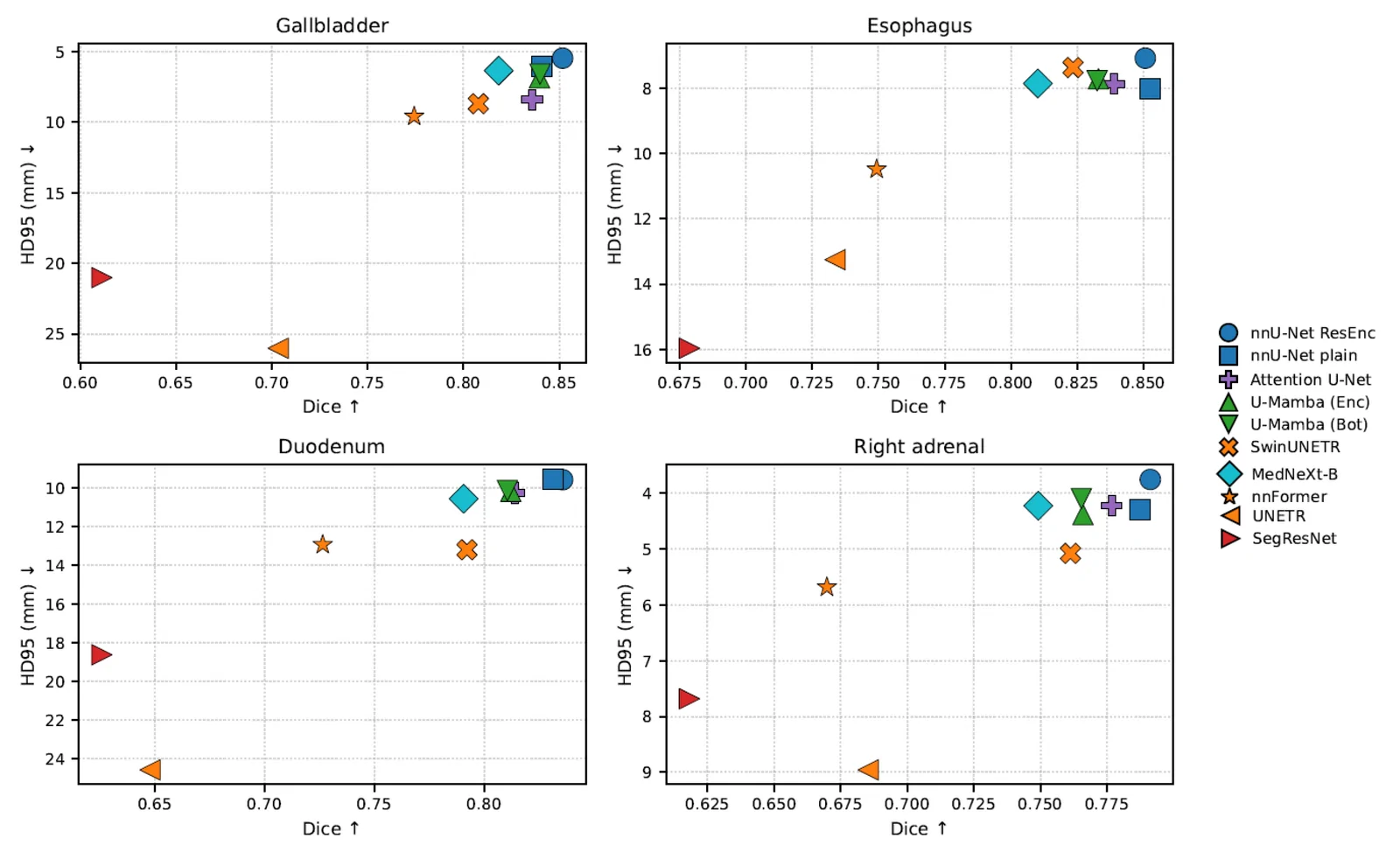
Dice versus HD95 on the four most variable organs. Mamba and CNN-attention models populate the top-right “safe” corner; SegResNet and UNETR sit in the bottom-left, “fails both” corner.

**Figure 7:**
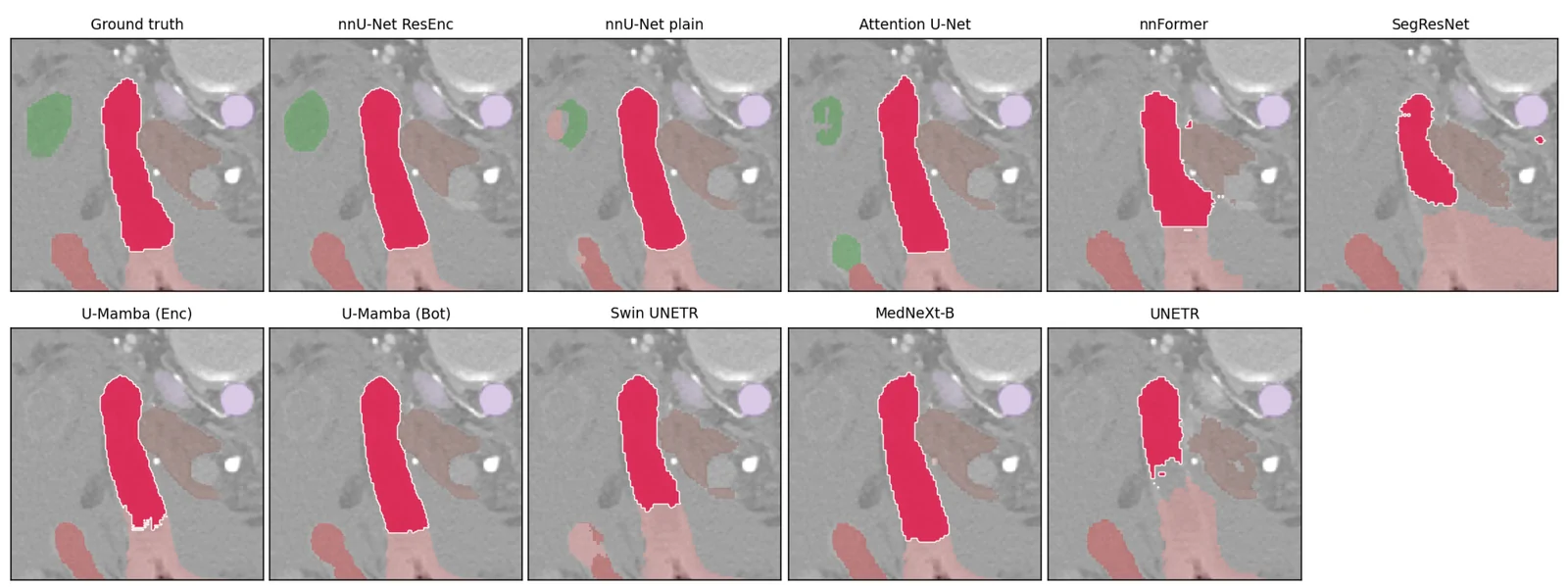
Single-case qualitative comparison on a fold-0 validation volume (amos_0242, axial slice z=164, the slice containing the most duodenum voxels). Panels, left-to-right and top-to-bottom: ground truth, nnU-Net ResEnc, nnU-Net plain, Attention U-Net, U-Mamba (Enc), U-Mamba (Bot), Swin UNETR, MedNeXt-B, nnFormer, UNETR, SegResNet. The duodenum is highlighted in red and the other AMOS organs are overlaid in faint grey. Predictions were resampled back to the original CT coordinate system. Four of ten models miss the gallbladder (the small structure above the duodenum) on this slice even though all ten recover the duodenum—a single-case illustration of the per-organ instability quantified in Table 6.

The four organs at the bottom right of the heatmap (gallbladder, oesophagus, duodenum, adrenal glands) are where architectures separate most clearly (Figure 6). Cross-model Dice spreads reach 24.1% on the gallbladder, 17.3–20.7% on the adrenal glands, and 18.8% on the duodenum, where even the leader manages only 83.6% Dice and 9.6 mm HD95 and UNETR collapses to 64.8% Dice/24.6 mm. Two organs have a genuinely contested ranking. On the oesophagus, a tubular structure, nnU-Net plain edges ResEnc by a thousandth on Dice, and every competitive model stays within HD95 7–8 mm. On the right adrenal gland, the Mamba variants trail the nnU-Net pair by 1–2% on Dice, plausibly because each gland fits inside the local receptive field of any 3D network, so Mamba’s long-range scan contributes no extra information. Figure 7 illustrates the same per-organ instability on a single case: every model recovers the duodenum on this slice, but four of ten miss the much smaller gallbladder lying on the same slice.

**Figure 8:**
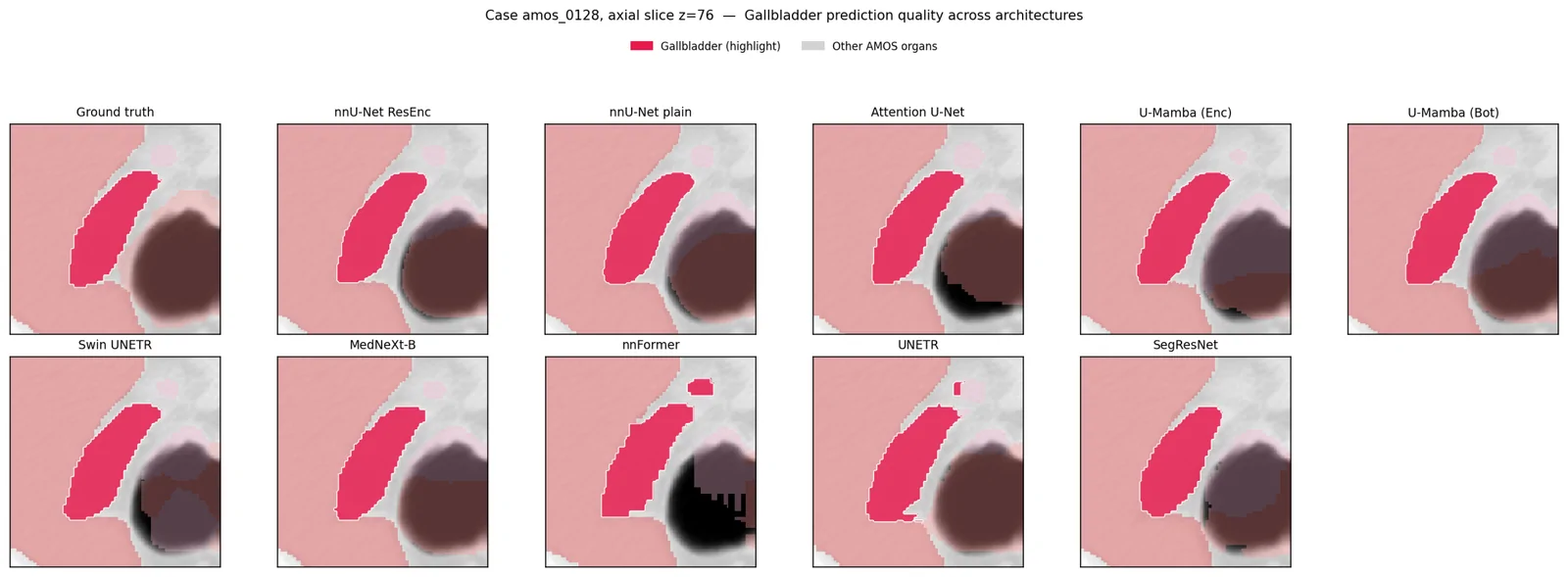
Qualitative gallbladder segmentation on a fold-0 validation case where every architecture succeeds (amos_0128, axial slice z=76). Panels, left-to-right and top-to-bottom: ground truth, nnU-Net ResEnc, nnU-Net plain, Attention U-Net, U-Mamba (Enc), U-Mamba (Bot), Swin UNETR, MedNeXt-B, nnFormer, UNETR, SegResNet. The gallbladder is highlighted in red and the other AMOS organs are overlaid in faint grey; predictions are resampled back to the original CT coordinate system. All ten architectures recover the organ (per-case gallbladder Dice 0.81–0.94), and the large organs visible in grey are segmented cleanly by every model—a representative successful case.

**Figure 9:**
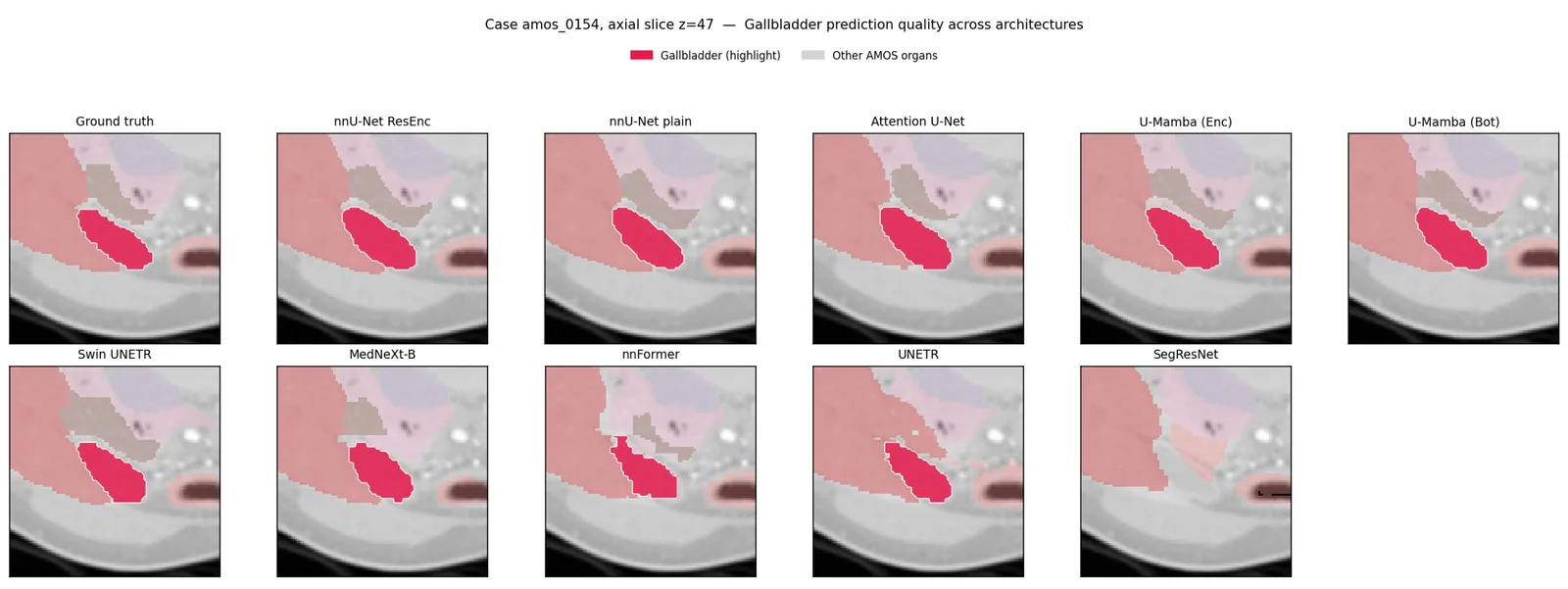
Qualitative gallbladder segmentation on a fold-0 validation case where SegResNet fails (amos_0154, axial slice z=47); panel order and overlay as in Figure 8. Nine of ten architectures recover the gallbladder, but SegResNet misses it entirely (per-case gallbladder Dice 0.04; on the same volume, it also collapses on the left adrenal gland at Dice 0.02), while the nnU-Net pair and the rest of the competitive cluster reach up to 0.92. This is the same organ, pipeline, and slice as shown in Figure 8, so the contrast is a property of the case and the architecture—the per-organ, per-case instability is quantified in Table 6.

To make the success/failure contrast explicit, Figure 8 and Figure 9 render the gallbladder, the single most variable structure in the benchmark (24.1% cross-model Dice spread), on two different validation cases, holding the pipeline, view, and highlight fixed. On a cleanly imaged case (Figure 8), all ten architectures recover the organ (per-case gallbladder Dice 0.81–0.94). On a harder case (Figure 9), nine models still recover it, but SegResNet misses it entirely (per-case Dice 0.04; the same volume also collapses its left adrenal gland to 0.02)—the small-organ failure that anchors SegResNet to the bottom of the ranking. Because the organ, pipeline, and slice are identical across the two figures, the difference is a property of the case and the architecture, not of organ difficulty. The large organs visible in faint grey (liver, stomach) are recovered by every model in both cases, so the three qualitative figures together span the size range: uniformly easy large organs, the mid-size duodenum (Figure 7), and the small gallbladder, where success is case- and architecture-dependent. This is the per-organ, per-case instability that a single aggregate Dice cannot express.

The transformer family is not internally consistent at the AMOS scale: Swin UNETR keeps a Mamba-level Dice on most small organs while UNETR collapses. The right statement is therefore that pure-ViT encoders are data-limited at this dataset size, while hierarchical window attention preserves enough locality to remain competitive.

### 4.7. External Validation on TotalSegmentator

To test the generalisability of the ten architectures beyond AMOS, we evaluated the fifty trained checkpoints, unchanged, as a five-fold ensemble per architecture, on 100 abdominal CT volumes from the independent TotalSegmentator dataset [16], remapped to the fifteen AMOS organs and preprocessed with the AMOS plans (zero-shot; no retraining or fine-tuning). Table 8 reports the per-organ Dice. Absolute Dice falls for every model under this domain and annotation shift: TotalSegmentator’s scans come from a different distribution than AMOS, and its labels are themselves model-generated rather than fully manual. Since this drop affects all architectures similarly, the informative quantity is not the absolute score but the relative ordering between models.

The ranking largely reproduces off-distribution: the Spearman rank correlation between the AMOS and TotalSegmentator overall-Dice orderings is ρ=0.84. The nnU-Net pair stays on top (0.834 and 0.832 external Dice) and the U-Mamba and MedNeXt convolutional models hold their positions (0.822–0.831). The clearest reshuffle is that the attention-based models transfer worst relative to their AMOS rank: Attention U-Net falls from third on AMOS (0.885) to seventh externally (0.756) and Swin UNETR drops from 0.871 to 0.770, whereas the planner topology CNNs and the state–space models are the most robust; SegResNet, UNETR and nnFormer stay at the bottom on both datasets.

Taking the external Dice leader, nnU-Net plain, as the reference, Table 9 reports the Holm–Bonferroni-corrected one-sided paired Wilcoxon *p*-values against each other model. Only nnU-Net ResEnc is statistically inseparable from nnU-Net plain overall (p=0.11; median per-case Dice difference with 0.05 points, and a win rate of 54%): the two nnU-Net variants form a genuine two-model top tie. U-Mamba (Enc) is the closest challenger (overall p=0.04, a 0.21-point median gap, not separable on 11 of the 15 organs), while every remaining architecture separates from the leader overall at $p<10^{-5}$. The effect sizes span three orders of magnitude, from 0.05 Dice points for ResEnc, through 0.7–1.4 points for the competitive band (MedNeXt-B, Attention U-Net, Swin UNETR), to 6–12 points for nnFormer, UNETR and SegResNet. The gaps lower in the ranking are both statistically significant and practically large.

## 5. Discussion

### 5.1. Principal Findings

Three findings emerge from the per-organ benchmark of ten architectures on AMOS CT. First, the top of the overall Dice ranking is a competitive cluster of convolutional and Mamba models: nnU-Net ResEnc, nnU-Net plain, the two U-Mamba variants, and MedNeXt-B all lie within about two Dice points, so the aggregate winner is not the only viable model. On the independent external set, the paired test finds nnU-Net ResEnc and nnU-Net plain statistically inseparable (Section 4.7). Second, the per-organ Dice ranking is stable on large organs (within 2% on the liver, the spleen, and the kidneys among the competitive top eight) and reshuffles by 10–13% on the small and geometrically complex ones (Figure 4, Table 6). Third, HD95 partitions the same ten architectures into three sharp tiers (Table 7) that do not match the Dice tiers. A model can be Dice-competitive and still produce clinically unacceptable contours. Both our results and metric theory support that HD95 carries information beyond Dice. Dice measures volumetric overlap and is insensitive to where errors fall. A compact boundary drift and a distant false-positive island can incur similar overlap penalties, whereas HD95 measures the robust upper tail of the surface–distance distribution in millimetres, so it responds to large contour deviations that barely move the overlap. Empirically, the two rankings diverge, with models that are close on aggregate Dice separating sharply on HD95 (Table 3 and Table 7). Therefore, comparable overlap does not imply comparable boundary fidelity. HD95 should accordingly be read as a complementary boundary-quality signal rather than a standalone clinical acceptance criterion.

### 5.2. Comparison with Prior Work

The most directly comparable study is nnU-Net Revisited [18], which benchmarked CNN, transformer, and Mamba families across six datasets including AMOS and identified the residual encoder nnU-Net as the aggregate winner. We reproduce that finding under an independent codebase, and add two qualifications. First, its top-1 status is shared by a competitive cluster within about two Dice points (nnU-Net plain, the two U-Mamba variants, MedNeXt-B), and it is statistically inseparable from nnU-Net plain on an independent external set (Section 4.7). Second, because the reported tables aggregate over the fifteen organs, they cannot resolve the per-organ reshuffle we report. The original AMOS paper [20] reports mean and surface Dice averaged over the fifteen classes for six baseline architectures; our ranking on the overlapping models matches theirs to within the noise of the training recipe. At the level of architecture paper claims, our results refine rather than contradict prior work. The transformer family is bimodal at AMOS scale: Swin [12] stays Dice-competitive but slips one HD95 tier, while UNETR [11] collapses on both metrics. U-Mamba’s selective-scan advantage [14] materialises mainly as boundary fidelity on large or elongated organs, without ever overtaking a CNN baseline at the top of any per-organ ranking.

### 5.3. Mechanistic Interpretation

The per-organ patterns are consistent with the inductive biases of each family. The nnU-Net pair benefits from a planner-derived topology that matches per-axis pooling depth to the data’s voxel spacing, giving the network an almost exact fit to abdominal CT anatomy. Deepening the encoder (ResEnc) does not change this bias, so its 0.4-point gain over the plain variant is negligible and statistically inseparable on external data (Section 4.7). Attention U-Net wins on NSD through a different mechanism: the decoder gates attenuate background activations near organ boundaries, suppressing spurious surface voxels that drive NSD penalties without inflating the Dice numerator. This gain does not transfer: on the external TotalSegmentator set, Attention U-Net drops from third to seventh (Section 4.7), suggesting its boundary gates are tuned to the AMOS distribution, whereas the planner topology CNNs and the state–space models generalise more robustly. The two U-Mamba variants gain HD95 on elongated or tubular structures where boundary localisation benefits from global context, but the gain disappears on the small adrenal glands, which fit inside any modern 3D receptive field. MedNeXt-B’s large-kernel ConvNeXt-3D design recovers most but not all of the nnU-Net topology benefit; the absence of a planner is the most plausible source of its residual gap on the long-tail organs (Table 6). The transformer family is bimodal: a pure-ViT encoder (UNETR) cannot learn the per-voxel locality prior from 300 CT cases and fails on topologically complex organs, while hierarchical window attention (Swin) and the local/global alternation of nnFormer recover Dice but not boundary fidelity. SegResNet, tuned originally for BraTS brain MRI [24], transfers poorly to heterogeneous abdominal CT intensities; this is a fixed-topology cross-domain failure, not a CNN failure, and it surfaces qualitatively as the complete loss of the gallbladder in Figure 9.

The parameter-count result is therefore best read as a statement about data-efficient inductive bias, not a rejection of model scaling. With only 300 labelled AMOS volumes and strong inter-organ class imbalance, extra parameters help only when the architecture also supplies the right priors (locality, multiscale context, anisotropic-resolution handling, and stable optimisation) which is why the compact nnU-Net variants, Attention U-Net, and MedNeXt-B stay competitive with, or beat, much larger transformers. The weaker UNETR and nnFormer results are correspondingly not evidence that transformers are unsuitable for medical segmentation, but that high-capacity models likely need stronger pre-training or more data before their capacity pays off. Architecture selection at this data scale should therefore turn on the interaction of capacity, inductive bias, and data efficiency, not on parameter count alone.

### 5.4. Implications for Clinical Use

The per-organ ranking has direct consequences for the three clinical applications introduced earlier. For radiotherapy planning of abdominal organs at risk [1], the relevant model-selection metric is HD95, since steep dose gradients turn a few-millimetre boundary error into a substantial dose error on adjacent tissue. On the long-tail OARs that drive plan complexity (adrenal glands, oesophagus, duodenum), only the nnU-Net pair stays inside a 5–10 mm HD95 band on all three. The U-Mamba variants follow within 1 mm, whereas UNETR and SegResNet (HD95 24.6 and 18.6 mm on the duodenum) are incompatible with current contour tolerances. The same boundary fidelity argument carries over to surgical planning [2], where the residual encoder nnU-Net is the lowest-HD95 model on the pancreas (5.4 mm), gallbladder (5.5 mm), and prostate/uterus (7.4 mm), and transformer-only encoders again fall outside routine surgical tolerances. Disease monitoring and longitudinal volumetry [3] depend on Dice instead: any top-six model is appropriate for liver and spleen volumetry (Dice ≥0.95), but for pancreas and adrenal follow-up only the nnU-Net pair and Attention U-Net remain reliable.

Three operational rules follow: (i) Aggregate Dice is not a sufficient model-selection criterion for any organ-specific clinical task. (ii) Where boundary fidelity drives outcome (dose conformality, surgical navigation, image-guided intervention), HD95 and NSD should be primary—not supplementary—acceptance metrics. (iii) Within the top competitive cluster (the convolutional and Mamba models within about two Dice points on AMOS; the nnU-Net pair statistically inseparable on external data), the right tie-breaker is the per-organ behaviour relevant to the intended clinical task. These rules are derived from retrospective benchmark comparisons rather than prospective clinical trials, and should inform model shortlisting rather than substitute for institution-specific validation prior to clinical use.

In decision terms, a radiotherapy pipeline should default to the nnU-Net pair, keeping the U-Mamba variants as an ensemble check when dose gradients are steep. A surgical-navigation pipeline should rank candidates by HD95 on the specific organ being navigated rather than by aggregate Dice, since a Dice-tied model can still be wrong for the one organ that matters. A longitudinal-volumetry pipeline tracking only the liver or spleen can take the cheapest of the top-six models, since scaling buys no accuracy on those saturated organs (Section 4.2).

### 5.5. Strengths and Limitations

The principal strengths of this work are methodological. All models share predefined nnU-Net-derived preprocessing, a common training and inference protocol, the same deterministic five-fold splits, and the same metric scripts; this reduces the recipe-tuning confounds called out in recent benchmarking literature [18] while still letting each architecture run at the input resolution its topology requires (Section 3.2). Pairwise significance is performed on an independent external set of fixed models evaluated on identical cases, rather than on cross-validated predictions that cannot be paired. To our knowledge, this is the first systematic per-organ benchmark on AMOS to combine boundary metrics, external validation, and a valid paired significance test.

Several limitations should be noted. The evaluation covers a single modality (CT). We assess cross-dataset generalization through the zero-shot TotalSegmentator validation of Section 4.7, which covers all fifteen organs and all ten architectures, but transfer to further centres and to the AMOS MRI subset remains untested. Fixed-topology models used isotropic preprocessing, whereas dynamic-topology models used anisotropic preprocessing (Section 3.2); consequently, comparisons between these two groups cannot be interpreted as purely architecture-only effects, since they also reflect each model’s compatible input resolution. We minimized this confound by using planner-derived configurations with comparable median voxel counts and by keeping the dataset splits, intensity normalisation, training recipe, inference protocol, and metric computation identical across all models. Each model used its published default hyperparameters; tuned variants might recover part of the gap, particularly for the data-hungry transformers. The Normalised Surface Dice used a uniform 1.0 mm tolerance across all fifteen organs; clinically-derived per-organ tolerances [30] would refine the comparison on small organs. Finally, all comparisons here are retrospective and no prospective clinical validation was performed; the architecture-selection guidance of Section 5.4 is therefore intended for model shortlisting under the conditions studied, not as evidence of clinical readiness.

### 5.6. Future Directions

Because this work is a comparative benchmark rather than a new model proposal, the main future value lies in testing whether the organ-level conclusions reported here are stable across datasets, modalities, metrics, and deployment settings. A separate study could take the following next steps: extending the external validation beyond TotalSegmentator to AbdomenAtlas under a comparable unified pipeline; cross-modality evaluation on the AMOS MRI subset to test whether the same architecture rankings and organ-specific failure modes transfer beyond CT; and per-organ specialist or mixture-of-experts segmentation networks motivated by the long-tail behaviour reported here.

Another direction concerns the recent shift towards promptable and foundation segmentation models. SAM-family adaptations to volumetric medical imaging (e.g., SAM-Med3D, MedSAM), together with interactive, prompt-driven variants of TotalSegmentator, raise a natural question. Could a single large pre-trained model, fine-tuned or prompted per organ, close the long-tail gap that separates the ten architectures benchmarked here, without the cost of training ten separate networks from scratch? Given that our results show the leaders’ advantage on small organs comes from architectural inductive bias rather than parameter count, whether a foundation model’s broader pre-training data can substitute for that bias on the adrenal glands, duodenum, and oesophagus is an open question. Future evaluations of such models should therefore use the same organ-stratified and boundary-aware protocol used here, rather than relying only on aggregate Dice. A related direction is to test whether the size-dependent inductive-bias effect reported here (Section 5.3) extends to architectures not evaluated in this benchmark, such as Vision Mamba variants and hybrid Transformer–Mamba designs that combine windowed attention with selective-scan blocks. These occupy a distinct point in the design space from the encoder-only U-Mamba variants tested here, and may recover some of the boundary fidelity gap seen for pure transformers (Section 4.5) without their full inference cost (Table 5).

Three further directions follow directly from the limitations noted in Section 5.5. First, adaptive or organ-weighted loss functions could be tested against the standard unweighted loss used here. For example, reweighting the Dice-cross-entropy loss per organ by inverse volume or by observed cross-fold variance. The question is whether they narrow the 10–13% Dice reshuffle on the adrenal glands, duodenum, and oesophagus (Section 4.4) without degrading the saturated large organs. Second, uncertainty quantification, for instance via test-time-augmentation variance, Monte Carlo dropout, or deep ensembles, would allow flagging low-confidence predictions on the long-tail organs identified here, which is particularly relevant given that these are the same organs where architecture rankings are least stable (Table 6). Third, clinically informed evaluation metrics (per-organ Normalised Surface Dice tolerances derived from inter-rater variability, rather than the uniform 1.0 mm tolerance) would refine the boundary fidelity comparison specifically on the small organs where a fixed tolerance is least representative of clinical acceptability.

A further direction is input restoration and adaptive activation functions for 3D segmentation. All ten backbones evaluated here use fixed activations (LeakyReLU or GELU). Whether a learnable, spatially-adaptive activation such as the SAGA operator of Siju et al. [40], originally proposed for 2D medical-image deblurring, improves boundary fidelity (HD95/NSD) on abdominal CT is an open question, as is the feasibility of restoration-aware preprocessing as a segmentation front-end.

## 6. Conclusions

We benchmarked ten state-of-the-art segmentation architectures on the AMOS CT split under an identical pipeline and reported per-organ Dice, HD95, and Normalised Surface Dice, with pairwise significance assessed on an independent external test set. The residual encoder nnU-Net is the aggregate winner, but it leads a competitive cluster of convolutional and Mamba models within about two Dice points. The ranking is stable on large organs (Dice spread under 3%) and reshuffles by 10–13% on the small or geometrically complex ones. HD95 partitions the same models into three sharp tiers that do not align with the Dice ranking, exposing a Dice-good/boundary-bad failure mode of UNETR and SegResNet that is invisible in any aggregate report. On an independent external set, the ranking largely reproduces (Spearman ρ=0.84), and a valid paired per-case test finds only nnU-Net plain and nnU-Net ResEnc statistically inseparable. The clinical takeaway is that aggregate Dice is not a sufficient model-selection criterion for any organ-specific clinical task: per-organ Dice and HD95 should be the primary acceptance metrics, with NSD added wherever boundary fidelity drives outcome.

## Figures and Tables

**Table 1:** Comparison of prior AMOS benchmarking studies with the present work, by evaluated architectures, reported metrics, dataset scope, and key limitation. Prior benchmarks rank architectures by a single aggregate Dice; this study adds per-organ stratification, boundary-sensitive metrics, a paired significance test, and external validation.

Study	Architectures Evaluated	Metrics	Dataset(s)	Key Limitation
AMOS benchmark (Ji et al. 2022) [20]	Six baselines: nnU-Net, V-Net, UNETR, Swin UNETR, CoTr, nnFormer	Mean Dice, NSD	AMOS CT and MRI	Aggregate mean over the fifteen organs; no per-organ significance testing or boundary-tier analysis
nnU-Net Revisited (Isensee et al. 2024) [18]	CNN, transformer and Mamba families	Mean Dice	Six datasets including AMOS CT	Reports one aggregate Dice per dataset; per-organ behaviour on AMOS undocumented
STU-Net (Huang et al. 2023) [32]	nnU-Net scaled from 14 M to 1.4 B parameters (one family)	Mean Dice	TotalSegmentator pre-training, transfer including AMOS	Single-family scaling and transfer study, not a cross-architecture per-organ comparison
**This work (2026)**	Ten models across four families under one identical pipeline	Per-organ Dice, HD95, NSD, precision, recall	AMOS CT and external TotalSegmentator	Adds per-organ and boundary-tier analysis, a paired external significance test, and external validation; limited to CT

**Table 2:** Summary of the ten architectures evaluated on AMOS. Parameter counts are measured from the trained checkpoint of fold 0 after deduplicating parameter aliases internal to the implementation.

Model	Family	Topology	Config	Params (M)
nnU-Net plain [9]	CNN U-Net	dynamic	fullres	31.1
nnU-Net ResEnc M [18]	CNN U-Net + residual encoder	dynamic	fullres	102.3
SegResNet [22]	Residual CNN + VAE branch	fixed	iso	87.2
Attention U-Net [25]	CNN with gated attention	fixed	iso	30.6
MedNeXt-B [27]	ConvNeXt-3D	fixed	iso	10.5
UNETR [11]	ViT encoder + CNN decoder	fixed	iso	121.4
Swin UNETR [12]	Hierarchical window attention	fixed	iso	63.1
nnFormer [13]	Interleaved local/global attn.	fixed	iso	151.2
U-Mamba (Bot) [14]	Residual U-Net + Mamba bottleneck	dynamic	fullres	41.9
U-Mamba (Enc) [14]	Residual U-Net + Mamba per encoder stage	dynamic	fullres	42.7

**Table 3:** Overall performance on AMOS CT. Each value is the mean ± standard deviation across the five validation folds. Models are sorted by mean Dice descending. Best per metric in bold.

Model	Dice ↑	HD95 (mm) ↓	NSD ↑
nnU-Net ResEnc	**0.893 ± 0.005**	**5.63 ± 0.38**	0.821 ± 0.006
nnU-Net plain	0.889 ± 0.006	5.97 ± 0.30	0.819 ± 0.004
Attention U-Net	0.885 ± 0.006	6.34 ± 0.78	**0.826 ± 0.009**
U-Mamba (Enc)	0.879 ± 0.008	6.15 ± 0.30	0.781 ± 0.013
U-Mamba (Bot)	0.879 ± 0.005	6.21 ± 0.41	0.779 ± 0.006
Swin UNETR	0.871 ± 0.008	9.30 ± 1.78	0.802 ± 0.011
MedNeXt-B	0.869 ± 0.005	6.54 ± 0.39	0.785 ± 0.008
nnFormer	0.825 ± 0.007	9.77 ± 0.40	0.718 ± 0.011
UNETR	0.800 ± 0.009	20.06 ± 2.05	0.669 ± 0.012
SegResNet	0.772 ± 0.013	17.46 ± 0.99	0.600 ± 0.015

**Table 4:** Additional aggregate metrics on AMOS CT, complementing Table 3: precision, recall (sensitivity), and the Jaccard index (IoU), foreground-averaged, mean ± standard deviation across the five validation folds; models sorted by Dice, which is repeated for reference. Best per column in bold.

Model	Dice ↑	Precision ↑	Recall ↑	IoU ↑
nnU-Net ResEnc	**0.893 ± 0.005**	**0.899 ± 0.007**	**0.900 ± 0.002**	**0.825 ± 0.006**
nnU-Net plain	0.889 ± 0.006	0.895 ± 0.010	**0.900 ± 0.005**	0.821 ± 0.007
Attention U-Net	0.885 ± 0.006	0.889 ± 0.009	0.896 ± 0.007	0.813 ± 0.007
U-Mamba (Enc)	0.879 ± 0.008	0.888 ± 0.009	0.885 ± 0.005	0.804 ± 0.010
U-Mamba (Bot)	0.879 ± 0.005	0.886 ± 0.006	0.887 ± 0.003	0.804 ± 0.006
Swin UNETR	0.871 ± 0.008	0.879 ± 0.011	0.882 ± 0.007	0.794 ± 0.010
MedNeXt-B	0.869 ± 0.005	0.875 ± 0.006	0.879 ± 0.007	0.789 ± 0.007
nnFormer	0.825 ± 0.007	0.837 ± 0.007	0.835 ± 0.013	0.730 ± 0.008
UNETR	0.800 ± 0.009	0.818 ± 0.009	0.810 ± 0.012	0.700 ± 0.011
SegResNet	0.772 ± 0.013	0.810 ± 0.012	0.772 ± 0.020	0.664 ± 0.015

**Table 5:** Computational efficiency of the ten architectures on AMOS CT, sorted by overall Dice (Table 3). **Params**: trainable parameters (Table 2). **GFLOPs**: floating-point operations of one forward pass on a single patch. **Infer.**: wall-clock of one full-volume sliding-window inference pass (tile overlap 0.5, Gaussian weighting, sliding-window batch two, no test–time augmentation) on a median-size AMOS volume. **Peak mem**: peak GPU memory during that pass. Compute figures are weight-independent and were measured on a single NVIDIA A100-SXM4-80 GB at each model’s AMOS input resolution. Training wall-clock is not reported.

Model	Params (M)	GFLOPs ↓	Infer. (s/vol) ↓	Peak Mem (GB) ↓
nnU-Net ResEnc	102.3	2346	2.67	5.1
nnU-Net plain	31.1	1265	1.84	4.7
Attention U-Net	30.6	782	1.86	4.7
U-Mamba (Enc)	42.7	2261 ^†^	8.61	12.4
U-Mamba (Bot)	41.9	2161 ^†^	4.04	5.2
Swin UNETR	63.1	1528	6.14	9.7
MedNeXt-B	10.5	335	4.99	5.7
nnFormer	151.2	849	2.67	4.3
UNETR	121.4	353	2.05	4.0
SegResNet	87.2	1303	2.13	4.0

^†^ U-Mamba GFLOPs are a lower bound: the attention-level FLOP counter cannot observe Mamba’s custom selective_scan CUDA kernel, so the true forward cost is higher.

**Table 6:** Per-organ Dice for the ten models on AMOS CT, fold-mean averaged across the five validation folds. Best per organ in bold. The cross-fold standard deviation is typically below 0.015 for the competitive cluster and rises to 0.02–0.05 on small organs for SegResNet and UNETR. The right-most column reproduces the overall mean of Table 3 for reference.

Model	Sp	RK	LK	GB	Es	Li	St	Ao	IVC	Pa	RAd	LAd	Du	Bl	Pr	Mean
nnU-Net ResEnc	**0.965**	**0.961**	0.956	**0.852**	0.851	**0.976**	**0.917**	**0.951**	0.912	**0.871**	**0.791**	0.798	**0.836**	**0.906**	**0.854**	**0.893**
nnU-Net plain	0.963	0.960	0.951	0.841	**0.853**	0.975	0.914	0.951	**0.912**	0.868	0.787	**0.798**	0.831	0.894	0.842	0.889
Attention U-Net	0.962	0.958	**0.957**	0.836	0.839	0.974	0.912	0.948	0.903	0.861	0.777	0.783	0.814	0.902	0.850	0.885
U-Mamba (Enc)	0.958	0.954	0.948	0.840	0.833	0.972	0.906	0.946	0.901	0.856	0.766	0.778	0.812	0.887	0.831	0.879
U-Mamba (Bot)	0.960	0.956	0.949	0.840	0.833	0.972	0.907	0.947	0.900	0.855	0.765	0.778	0.811	0.882	0.831	0.879
Swin UNETR	0.955	0.950	0.946	0.808	0.824	0.970	0.892	0.945	0.896	0.845	0.761	0.773	0.792	0.883	0.829	0.871
MedNeXt-B	0.952	0.953	0.950	0.818	0.810	0.969	0.893	0.944	0.892	0.842	0.749	0.761	0.791	0.879	0.829	0.869
nnFormer	0.944	0.939	0.932	0.774	0.749	0.962	0.870	0.922	0.851	0.795	0.670	0.671	0.727	0.818	0.756	0.825
UNETR	0.935	0.918	0.914	0.703	0.734	0.953	0.808	0.921	0.837	0.756	0.685	0.669	0.648	0.781	0.735	0.800
SegResNet	0.904	0.907	0.908	0.611	0.679	0.939	0.804	0.912	0.817	0.743	0.618	0.591	0.626	0.775	0.743	0.772

Sp, spleen; RK, right kidney; LK, left kidney; GB, gallbladder; Es, oesophagus; Li, liver; St, stomach; Ao, aorta; IVC, inferior vena cava; Pa, pancreas; RAd, right adrenal gland; LAd, left adrenal gland; Du, duodenum; Bl, bladder; Pr, prostate/uterus.

**Table 7:** Per-organ HD95 in millimetres for the ten models on AMOS CT, fold-mean averaged across the five validation folds. Lower is better; best per organ in bold.

Model	Sp	RK	LK	GB	Es	Li	St	Ao	IVC	Pa	RAd	LAd	Du	Bl	Pr	Mean
nnU-Net ResEnc	2.5	4.0	5.9	**5.5**	**7.1**	**4.1**	8.8	5.2	3.7	**5.4**	**3.8**	**4.3**	9.6	**7.2**	**7.4**	**5.63**
nnU-Net plain	**2.5**	4.5	4.3	6.1	8.0	5.2	**8.3**	5.5	3.9	5.9	4.3	4.3	**9.6**	7.4	9.7	5.97
U-Mamba (Enc)	3.7	3.8	5.6	6.9	7.7	5.3	9.3	4.8	4.3	6.2	4.4	4.8	10.2	7.5	7.7	6.15
U-Mamba (Bot)	2.6	**3.5**	4.8	6.6	7.8	5.3	9.9	5.7	4.3	6.1	4.1	4.7	10.2	8.1	9.5	6.21
Attention U-Net	3.9	4.7	4.0	8.4	7.9	5.9	9.9	**4.0**	**3.6**	6.2	4.2	5.1	10.3	7.6	9.4	6.34
MedNeXt-B	4.3	4.6	**3.6**	6.4	7.9	7.4	11.3	4.8	4.0	6.8	4.2	4.8	10.6	7.7	9.9	6.54
Swin UNETR	7.2	7.6	15.9	8.7	7.4	7.5	19.3	4.8	4.8	7.8	5.1	5.6	13.2	11.0	14.0	9.30
nnFormer	6.9	9.0	10.5	9.6	10.5	7.9	14.3	7.6	6.1	9.2	5.7	6.9	12.9	13.6	15.9	9.77
UNETR	16.9	29.3	35.0	26.0	13.3	13.9	30.5	7.8	10.0	19.6	9.0	13.8	24.6	22.7	28.6	20.06
SegResNet	18.0	27.3	25.2	21.0	16.0	25.2	29.5	6.7	9.4	13.9	7.7	9.4	18.6	14.9	19.2	17.46

Column abbreviations as in Table 6. The **Mean** column is the fifteen-organ average of the HD95 means.

**Table 8:** Zero-shot per-organ Dice on 100 external TotalSegmentator CT volumes, for the AMOS-trained five-fold ensembles (no retraining). Rows are ordered by AMOS overall Dice; the Spearman correlation between the AMOS and external overall-Dice rankings is ρ=0.84. Column abbreviations as in Table 6.

Model	Sp	RK	LK	GB	Es	Li	St	Ao	IVC	Pa	RAd	LAd	Du	Bl	Pr	Overall
nnU-Net ResEnc	0.932	0.921	0.917	0.738	0.828	0.964	0.914	0.918	0.889	0.865	0.777	0.802	0.735	0.856	0.422	0.832
nnU-Net plain	0.938	0.922	0.922	0.732	0.839	0.971	0.914	0.921	0.893	0.867	0.781	0.804	0.736	0.854	0.423	0.834
Attention U-Net	0.870	0.836	0.832	0.697	0.771	0.912	0.836	0.856	0.813	0.746	0.649	0.678	0.659	0.782	0.407	0.756
U-Mamba (Enc)	0.933	0.917	0.934	0.795	0.813	0.969	0.908	0.916	0.887	0.860	0.764	0.790	0.737	0.833	0.413	0.831
U-Mamba (Bot)	0.911	0.911	0.937	0.767	0.801	0.964	0.901	0.913	0.886	0.844	0.755	0.793	0.732	0.826	0.386	0.822
Swin UNETR	0.861	0.902	0.907	0.657	0.756	0.896	0.822	0.889	0.834	0.778	0.692	0.735	0.673	0.784	0.368	0.770
MedNeXt-B	0.907	0.914	0.923	0.746	0.804	0.968	0.886	0.912	0.887	0.835	0.771	0.794	0.735	0.837	0.404	0.822
nnFormer	0.838	0.841	0.819	0.633	0.702	0.925	0.826	0.848	0.786	0.685	0.609	0.629	0.636	0.704	0.241	0.715
UNETR	0.832	0.867	0.849	0.683	0.709	0.919	0.770	0.887	0.828	0.638	0.698	0.696	0.583	0.754	0.286	0.733
SegResNet	0.798	0.839	0.814	0.594	0.632	0.901	0.803	0.866	0.799	0.665	0.533	0.503	0.579	0.723	0.349	0.693

**Table 9:** Pairwise corrected one-sided paired Wilcoxon *p*-values on the external TotalSegmentator set: nnU-Net plain (the external Dice leader) vs. each of the nine other architectures, on per-case per-organ Dice, over the 100 shared cases. Holm–Bonferroni correction across the fifteen organs within each row; the right-most column is the test on the per-case fifteen-organ mean Dice (no within-row correction). Cells show the significance band only: *** *p* < 0.001, ** p<0.01, * p<0.05, n.s. otherwise. Column abbreviations as in Table 6.

Challenger	Sp	RK	LK	GB	Es	Li	St	Ao	IVC	Pa	RAd	LAd	Du	Bl	Pr	Overall
nnU-Net ResEnc	n.s.	*	n.s.	n.s.	*	n.s.	n.s.	n.s.	n.s.	n.s.	n.s.	n.s.	n.s.	**	n.s.	n.s.
Attention U-Net	n.s.	n.s.	n.s.	n.s.	***	n.s.	n.s.	***	**	**	***	***	n.s.	n.s.	n.s.	***
U-Mamba (Enc)	n.s.	n.s.	n.s.	n.s.	***	n.s.	n.s.	*	n.s.	***	n.s.	***	n.s.	n.s.	n.s.	*
U-Mamba (Bot)	*	*	n.s.	n.s.	***	*	n.s.	*	n.s.	***	***	***	n.s.	***	**	***
Swin UNETR	**	***	**	**	***	***	***	***	***	***	***	***	***	***	**	***
MedNeXt-B	***	***	**	n.s.	***	*	n.s.	*	n.s.	***	n.s.	*	n.s.	n.s.	n.s.	***
nnFormer	***	***	***	***	***	***	***	***	***	***	***	***	***	***	***	***
UNETR	***	***	***	***	***	***	***	***	***	***	***	***	***	***	***	***
SegResNet	***	***	***	***	***	***	***	***	***	***	***	***	***	***	***	***

## Data Availability

The data presented in this study are openly available in AMOS22 at https://amos22.grand-challenge.org/ (accessed on 8 April 2026).

## Abbreviations

The following abbreviations are used in this manuscript:

AMOS	Abdominal Multi-Organ Segmentation
CNN	Convolutional Neural Network
CT	Computed Tomography
CV	cross-validation
DSC	Dice Similarity Coefficient
HD95	95-percentile Hausdorff Distance
HU	Hounsfield Unit
MoE	Mixture of Experts
MRI	Magnetic Resonance Imaging
NSD	Normalised Surface Dice
OAR	Organ At Risk
SGD	stochastic gradient descent
SSM	state–space model
TTA	test–time augmentation
VAE	variational autoencoder
ViT	Vision Transformer
